# Proteostasis collapse, inter-tissue communication, and the regulation of aging at the organismal level

**DOI:** 10.3389/fgene.2015.00080

**Published:** 2015-03-05

**Authors:** Tatyana Dubnikov, Ehud Cohen

**Affiliations:** Department of Biochemistry and Molecular Biology, The Institute for Medical Research Israel – Canada, The Hebrew University School of MedicineJerusalem, Israel

**Keywords:** aging, proteostasis, hyper-function, insulin-IGF signaling, stress-resistance

What is the nature of the molecular and cellular mechanisms that drive the aging process is a puzzling unsolved enigma that has been under debate for decades. Several prominent theories have been proposed to explain the mechanistic basis of aging. Late in the nineteenth century, the German biologist August Weismann noticed that the lifespans of different species, which live in similar environments, differ greatly. Based on this observation he predicted that the duration of an animal's life is set by internal organismal components rather than external factors (Weismann, 1889). In modern terms this theory suggests that the pace of aging is governed by the activity of biological mechanisms such as signaling cascades that control the expression of gene networks, post-translational modifications or epigenetic factors. The rationale underlying this theory suggests that aging is a regulated, programmed process which is amenable to the activity of specialized mechanisms.

The idea that aging is a regulated process has been challenged by alternative models which proposed that aging results from a constant accumulation of molecular damage that impairs cellular functions and leads to functional decline. According to this theory, aging-promoting damage ensues from the formation of hazardous metabolic byproducts such as reactive oxygen species (ROS) (Harman, 1956) which attack macromolecules. Later it was proposed that ROS are released by mitochondrial respiration (Harman, 1972). The damage accumulation theory proposes that aging is driven by stochastic events and thus, progresses randomly.

The postulation that genes regulate the aging process prompted researchers to search for mutations that extend lifespan. Several long-lived strains of the nematode *Caenorhabditis elegans* (*C. elegans*) were isolated by Michael Klass who exposed worms to chemical mutagenesis and recorded the lifespans of their offspring (Klass, 1983). Later, the Johnson laboratory further characterized these strains and found that one strain carries a mutation in a kinase, which they termed “*age-1*,” and exhibits mean lifespan of approximately 40% longer than the mean lifespan of their wild-type counterparts (Friedman and Johnson, 1988). This finding provided an apparent indication that indeed, aging is regulated by gene products. Nevertheless, the authors reported in the same article that the lifespan variations within isogenic worm populations are not hereditable, proposing that the duration of an individual animal's life is concurrently amenable to stochastic events and regulatory mechanisms.

Over the years, mutations in numerous genes and metabolic manipulations have been found to affect lifespans of different animals. “*age-1*” was found to be a component of the insulin/insulin-like growth factor 1 (IGF-1) signaling (IIS) cascade. Reducing the activity of this highly conserved pathway by RNA interference (RNAi) or mutation extends lifespans of worms (Kenyon et al., 1993), flies (Giannakou and Partridge, 2007), mice (Holzenberger et al., 2003), and probably humans (Suh et al., 2008). The IIS negatively regulates the activity of key transcription factors that govern the expression of gene networks which modulate aging and lifespan (Lee et al., 2001; Hsu et al., 2003; Tullet et al., 2008), mediate resistance to various stress conditions (Murakami and Johnson, 1996; Honda and Honda, 1999) and preserve proteostasis of worms (Morley et al., 2002; Cohen et al., 2006) and mice (Cohen et al., 2009; Freude et al., 2009). Similarly, the lifespan extension that emanates from reduced food intake (McCay et al., 1989 “dietary restriction” or “DR”) is dependent upon the activity of transcription factors (Bishop and Guarente, 2007; Panowski et al., 2007) that mediate stress resistance and promote improved proteostasis (Steinkraus et al., 2008). The ablation of germ cells is an additional manipulation that extends lifespan (Hsin and Kenyon, 1999) in a transcription factors-dependent manner (Berman and Kenyon, 2006) and confers proteostasis robustness (Shemesh et al., 2013). Reduction in the activity of the mitochondrial electron transport chain (ETC) was also identified as an interference that extends lifespan (Feng et al., 2001; Dillin et al., 2002).

Collectively, the discoveries that the manipulation of different biological mechanisms extends lifespan and slows the progression of aging-associated phenotypes, such as proteostasis deterioration and stress sensitivity, supported the idea that aging has regulated aspects which are programmed within the organism. But to what extent and how these mechanisms regulate the aging process remained largely obscure.

A possible explanation proposes that the aforementioned, aging-altering metabolic manipulations increase the organism's ability to resist environmental insults, thereby reducing the rate of damage accumulation. Indeed, subsets of genes that protect from stress were identified as targets of aging-controlling pathways.For example, IIS reduction elevates the expression levels of members of the super oxide dismutase (*sod*) gene family as well as of catalase (Murphy et al., 2003), which are known to detoxify ROS. Similarly, IIS reduction elevates the expression levels of genes that encode molecular chaperones that play key roles in the maintenance of proteostasis (Murphy et al., 2003). DR also induces the expression of certain *sod* family members (Panowski et al., 2007). These correlations suggested that aging-regulating pathways slow aging by elevating the organism's ability to resist stress conditions and by promoting the maintenance of proteostasis; however, the idea that sensitivity to stress conditions promotes aging has been seriously challenged by several recent studies. First, the deletion of all five *sod* genes hyper-sensitized worms to oxidative stress but did not affect the lifespans of unstressed animals (Van Raamsdonk and Hekimi, 2012). In addition, stress resistance and lifespan are not necessarily coupled (Maman et al., 2013; Volovik et al., 2014). Finally, the exposure of nematodes to low ROS concentrations increased their lifespan suggesting that these molecules serve as messengers that can slow the progression of aging (Lee et al., 2010; Yang and Hekimi, 2010). These findings imply that accumulation of oxidation-mediated damage cannot be the sole driving force behind the aging process.

If not beneficial for stress resistance, why would animal harbor genes that promote aging and how such genes contribute to the organism's fitness? An early theory, known as the “antagonistic pleiotropy hypothesis” proposes that some genes encode proteins that are advantageous early in life but deleterious in late life stages. In such case, the natural selection process is expected to favor benefit early in life despite the late-life damage (Williams, 1957). Recently, an additional theory, which doubts the concept that aging is a programmed process, has been proposed. This hypothesis, which was named the “hyper-function theory,” elaborates on the trade-off theory of Williams (1957) suggesting that since the chances of a wild animal to reach old age are slim, as it will most probably die due to external events (such as predation or infection), it is irrational for the organism to invest its limited resources in maintenance. Thus, aging results from a set of maladies that emerge as a result of loose regulation of pathways that are crucial for growth, maturation and reproduction early in life but cause damage in late life stages (Blagosklonny, 2006). This model implies that the inhibition of these hyper-functioning pathways reduces the rate of damage accumulation and slows the rate of aging. An example of such mechanism is the production of yolk in the nematode *C. elegans*, which plays important roles during reproduction but causes damage in post-reproductive stages. Interestingly, yolk production is reduced by IIS inhibition, correlating the hyper-function of this mechanism to the aging-controlling features of the IIS pathway (reviewed in Gems and Partridge, 2013). The hyper-function theory proposes that the aging process is not programmed but results from a gradual accumulation of damage. Although this theory provides explanations to some of the open questions in the field (Blagosklonny, 2007), it fails to explain key observations. If the hyper-function of the IIS causes damage in late stages of life it is predicted that its activity would affect lifespan solely during adulthood, after the animal completed development. Nevertheless, the heat shock factor 1 (HSF-1), a transcription factor that is required for IIS reduction-mediated lifespan extension (Hsu et al., 2003) is foremost needed for longevity assurance during larval development (Volovik et al., 2012). This study indicates that IIS reduction also affects lifespan and aging early in life, during development.

Recent developments in the research of aging further challenge the hyper-function theory. If aging was solely the result of deleterious hyper-function in late stages of life, it would be expected that proteostasis would gradually deteriorate in post reproductive stages. *De facto*, it was found that in *C. elegans*, proteostasis collapses within a very narrow time window right after the animal's transition to adulthood and entry to the reproductive period (Ben-Zvi et al., 2009; Labbadia and Morimoto, 2014), when according to the theory it should exhibit optimal functionality. Moreover, neglected hyper-function is not expected to be actively controlled by inter-tissue communication. However, the lifespans of nematodes are regulated by neuronal signaling mechanisms that orchestrate the activity of aging-governing mechanisms in distal tissues. Neuronal signaling mechanisms were found to plays key roles in ETC-mediated longevity (Durieux et al., 2011), DR-controlled lifespan extension (Bishop and Guarente, 2007) and in the activation of IIS-regulated transcription factors (Zhang et al., 2013). Furthermore, the hyper-activation of the endoplasmic reticulum's unfolded protein response (UPR^ER^)—regulating transcription factor XBP-1 in neurons, activates this mechanism in distal tissues and extends lifespan rather than causing lifespan-shortening damage (Taylor and Dillin, 2013).

So, what are the driving forces behind the aging process? Is it a regulated or stochastic process? The insights that were obtained during decades of aging research strongly suggest that aging is a multifaceted process which is driven by a nexus of mechanisms and probably cannot be explained by a single theory. Some aspects of aging are clearly stochastic, explaining the variability in lifespans within populations. Other aspects are regulated in cell-autonomous and non-autonomous manners, underlying the orchestration of proteostasis at the organismal level and the extension of lifespan by the activation of neuronal components. Additional aspects may stem from the hyper-function of developmental pathways.

## Conflict of interest statement

The authors declare that the research was conducted in the absence of any commercial or financial relationships that could be construed as a potential conflict of interest.
